# First Principle Study on the Effect of Strain on the Electronic Structure and Carrier Mobility of the Janus MoSTe and WSTe Monolayers

**DOI:** 10.3390/nano13182535

**Published:** 2023-09-11

**Authors:** Jawad El Hamdaoui, Laura M. Pérez, Miguel Ojeda-Martínez, Nassima El Ouarie, Pablo Díaz, David Laroze, El Mustapha Feddi

**Affiliations:** 1 Laboratory of Condensed Matter and Interdisciplinary Sciences (LaMCScI), Faculty of Sciences Rabat, Mohammed V University in Rabat, Rabat 10000, Morocco; jawad.elhamdaoui@um5r.ac.ma (J.E.H.);; 2 Group of Optoelectronic of Semiconductors and Nanomaterials, ENSET of Rabat, Mohammed V University in Rabat, Rabat 10000, Morocco; 3 Departamento de Física, Universidad de Tarapacá, Casilla 7D, Arica 1000000, Chile; lperez@academicos.uta.cl; 4 Centro Universitario de los Valles, Universidad de Guadalajara, Carretera Guadalajara-Ameca, Ameca 46600, Jalisco, Mexico; 5 Departamento de Ciencias Físicas, Universidad de La Frontera, Casilla 54-D, Temuco 4780000, Chile; 6 Instituto de Alta Investigación, Universidad de Tarapacá, Casilla 7D, Arica 1000000, Chile; 7 Institute of Applied Physics, Mohammed VI Polytechnic University, Lot 660, Hay Moulay Rachid, Ben Guerir 43150, Morocco

**Keywords:** DFT, 2D Janus, carrier mobility, electronic structure, MoSTe, WSTe

## Abstract

Using first-principle calculations, we investigate the impact of strain on the electronic structures and effective masses of Janus WSTe and MoSTe monolayers. The calculations were performed using the QUANTUM-ESPRESSO package, employing the PBE and HSE06 functionals. Our results demonstrate that strain fundamentally changes the electronic structures of the Janus WSTe and MoSTe monolayers. We observe that deformation causes a shift in the maxima and minima of the valence and conduction bands, respectively. We find that the effective electrons and hole masses of MoSTe and WSTe can be changed by deformation. In addition, the strain’s effect on carrier mobility is also investigated in this work via the deformation potential theory.

## 1. Introduction

Two-dimensional (2D) materials have received much attention from the scientific community. In 2D materials, electrons are confined in two spatial directions, which implies that the quantum confinement effect could modify the electronic, vibrational, and optical properties. These properties could differ from their bulk counterparts [1,2,3], opening up the possibility of using 2D nanomaterials to create new devices, which could be used in areas such as electronics, spintronics, optoelectronics, energy conversion, and more. For example, the large surface area of 2D materials allows them to be used in sensors, light detectors, and supercapacitors, which means that 2D material applications encompass everything from batteries and electrocatalysis to electronics and photonics [3,4,5]. Some promising low-dimensional materials are monolayers based on transition metal dichalcogenides (TMDs), as reported previously by [1,6,7]. It is well known that TMD monolayers are formed by transition metal atoms (M) from groups IV to VII, and two chalcogen atoms (X), such as S, Se, and Te [8,9]. The chemical formula corresponds to MX${}_{2}$, representing materials such as MoS${}_{2}$, MoTe${}_{2}$, MoSe${}_{2}$, WS${}_{2}$, and WSe${}_{2}$. These materials have been theoretically studied and synthesized experimentally [2,4,6,7,10]. Even though bulk TMDs have indirect band gaps, the size reduction changes the band’s structural behavior from an indirect to a direct band gap. Also, in accordance with some authors, the band gaps range from 1.0 to 2.0 eV [9,11]. This means that these materials could be employed in applications for optoelectronic, sensing, or solar energy; due to these promising applications, some efforts have been made to improve the properties of 2D TMD materials. For example, Mahdi Ghorbani-Asl et al. synthesized TMD nanotubes, and the results of their studies demonstrate that the electronic properties of TMD nanotubes depend on (and are controllable by) the application of a tensile strain [12]. In another work, Tatiana V. Shubina reported that TMD nanotubes formed by a dozen monolayers have indirect band gaps contributing to photoluminescence emissions [13].

Other studies have demonstrated that TMD quantum dots could be useful in electronics and biomedical applications [14,15]. Furthermore, if 2D TMD materials have different compositions—one on the top layer and the other on the bottom layer—this is referred to as the Janus TMD. Some examples are MoSSe, MoSTe, WSTe, and WSeTe. Janus TMDs are represented by MXY. In particular, WXY and MoXY have band gaps in the range of 0.16–1.91 and 0.94–1.69 eV, respectively [1,9,16]. The band gap values imply that the Janus TDM monolayer could also be used in optoelectronics. In particular, some interesting results have been found. For example, the report by Yagmurcukardes et al. suggests that as a result of an electron doping strategy, the electron mobility increases up to $1.21\times 10^{5}$$\frac{{\mathrm{cm}}^{2}}{V.s}$ (and for hole, $7.24\times 10^{4}$$\frac{{\mathrm{cm}}^{2}}{V.s}$) [1]. Also, the difference in electronegativity from the atoms on each side of the layers could increase the exciton lifetime with the possibility of being used in light detection and harvesting [2,11]. As we can see, the TMD monolayers have a lot of promising applications. Still, we need to perform more theoretical and experimental research to understand the properties of the Janus TMD monolayers.

Strain engineering is a powerful technique that involves applying strain to a material to modify its properties. In the case of 2D materials, strain directly affects the lattice structure, leading to alterations in the electronic properties of the material. This, in turn, influences various characteristics, such as the band gap, thermal conductivity, and chemical reactivity.

Various approaches exist for strain engineering in 2D materials. One method involves lithographic patterning [17] of stressed thin films to selectively induce tension, compression, uniaxiality, and biaxiality relative to crystal axes. Additionally, novel experimental methods have been developed to overcome the limitations of traditional bulk mechanical testing for atomically thin structures [18,19].

Theoretical research predicts that a large strain induces significant polarization in 2D materials. Furthermore, the strain influences the chemical reactivity of 2D materials, such as monolayer MoS2, which shows promising catalytic behavior in the hydrogen evolution reaction (HER) under the strain [20].

In this study, we explore the effect of strain on the electronic structure and carrier mobility of Janus MoSTe and WSTe monolayers. We employ first-principle calculations and consider the influence of strain on these materials.

This paper is structured as follows: In Section 2, we present a comprehensive overview of our calculation’s methodology. Section 3 encompasses the results obtained from our calculations, focusing on the electronic properties and the impact of strain on carrier mobility. Finally, in Section 4, we provide a concise conclusion, summarizing the key findings of our study.

## 2. Computational Method

In this work, we analyze the electronic band structure of the Janus TMD monolayers of MoSTe and WSTe by the density functional theory (DFT). Using the projector augmented wave (PAW) approach, the Quantum ESPRESSO package was employed for all DFT computations [21]. The electronic exchange–correlation energy was treated by the generalized-gradient approximation of Perdew–Burke–Ernzerhof (GGA-PBE) and hybrid functionals. We utilize the Heyd–Scuseria–Ernzerhof (HSE06) functional for the hybrid functional, where the exchange and correlation potential combine the PBE functional and the Hartree–Fock approach [22]:

(1) $$E_{xc}^{HSE}=\alpha .E_{x}^{HF,SR}+\left(1-\alpha \right)E_{x}^{PBE,SR}+E_{x}^{PBE,LR}+E_{c}^{PBE},$$

where $E_{x}^{PBE,SR}$ and $E_{x}^{LR,PBE}$ are the long-range (LR) and short-range (SR) components of the PBE exchange energies, which are range-separated to reduce the computing cost of the PBE exchange, and $E_{c}^{PBE}$ is the correlation energy functional of the generalized gradient approximation (GGA-PBE). $E_{x}^{SR,HF}$ denotes the short range of the Hartree–Fock energy. The mixing parameter alpha is fixed in the original HSE functional (HSE06) to 0.25, but it can be adjusted in some cases to match the experimental results. In our study, we selected the default value of alpha (0.25) in the HSE06 functional, as it is widely used and has been shown to provide reasonable results for various systems. This alpha choice was based on its common usage and the potential to yield reasonable predictions for the band gap energy in Janus MoSTe and WSTe monolayers. In the computational simulations, rigorous convergence tests were conducted to ensure accurate and reliable results. To represent the wave function, a cut-off energy of 80 Ry (Rydberg) was carefully chosen, guaranteeing that the calculations reached the desired level of precision. To accurately sample the Brillouin zone for all Janus transition metal dichalcogenides (TMDs), an 18 × 18 × 1 k-point grid mesh was employed. This dense mesh ensured a thorough and precise exploration of the electronic band structure and other important properties. To eliminate any unwanted interactions between adjacent slabs in the simulation, a vacuum layer with a thickness of 28 Å was introduced in the z-direction. This separation prevented any spurious effects from the interaction between the neighboring layers and ensured that the simulation accurately represented the isolated properties of the Janus TMDs. To obtain the most stable and energetically favorable structures of the Janus TMDs, the Broyden–Fletcher–Goldfarb–Shanno (BFGS) algorithm was employed. This optimization algorithm efficiently minimized the total energy of the system while adjusting the lattice parameters accordingly, resulting in the relaxed lattice parameters that best represented the real-world characteristics of the Janus TMDs [23,24].

**Figure 1:**
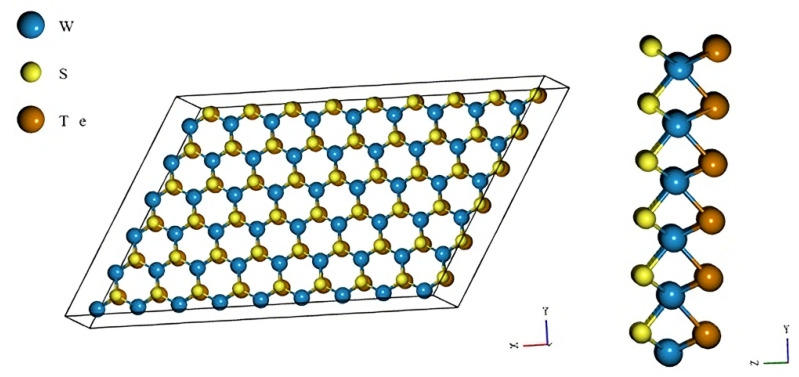
Schematic representation of the Janus TMD monolayer (WSTe).

In Figure 1, we presented a schematic representation of the Janus TMD monolayer; for example, we presented the WSTe monolayer. As seen in Figure 1, one face of the monolayer is composed solely of S atoms. In contrast, the other face of the monolayer is composed of Te atoms, while the atoms at the center of the monolayer are W (or Mo in the case of MoSTe).

## 3. Results and Discussion

**Figure 2:**
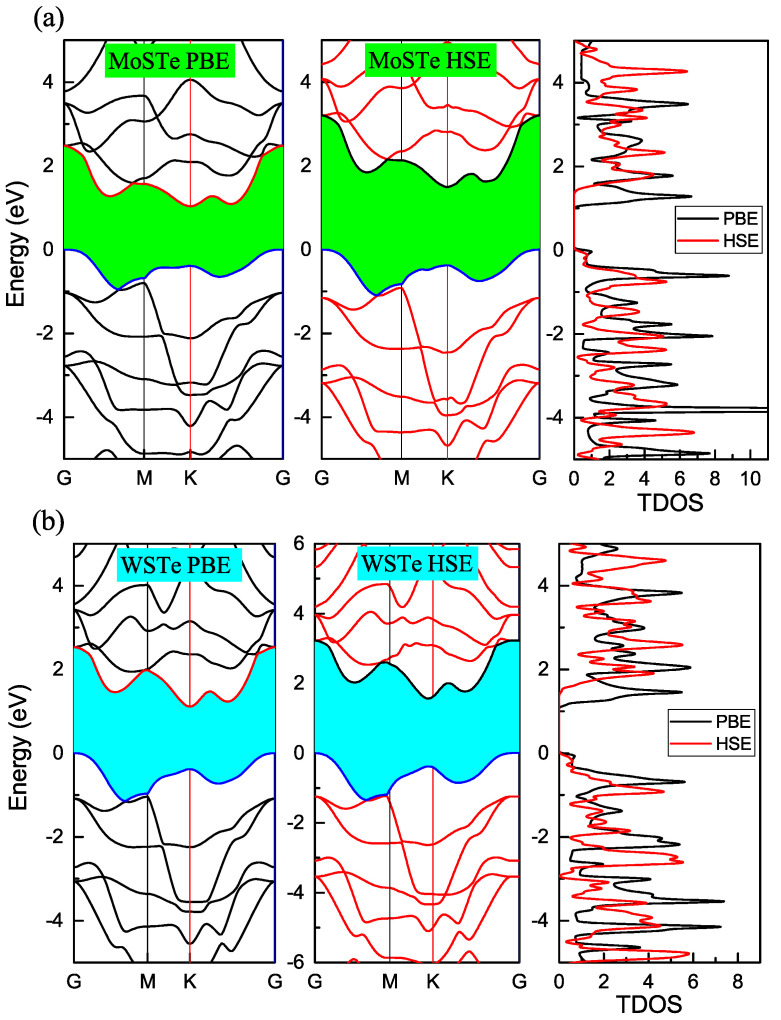
(**a**,**b**) Energy band gap and total density of states of WSTe and MoSTe using GGA-PBE and HSE06 functionals.

In Figure 2a,b, we present the electronic band structures of MoSTe and WSTe using two different density functionals: PBE and HSE06.

Both PBE and HSE06 functionals successfully capture the indirect behaviors of the band structures in MoSTe and WSTe monolayers. These behaviors have been previously reported in studies, such as in [1,10,16,25]. These references show that MoSTe and WSTe are indirect band gap semiconductors. In their electronic structures, the valence band maximum is located at the Gamma point, while the conduction band minimum is situated at the K point of the first Brillouin zone. As reported in [26,27], the band gap energies are approximately 1.17 eV for MoSTe and 1.35 eV for WSTe.

In our GGA-PBE functional calculations, we obtained band gap values of 1.03 eV for MoSTe and 1.10 eV for WSTe. These values show discrepancies of approximately 12% and 18.5% when compared to the results reported in other papers. It is important to note that the GGA-PBE functional, while accurately reproducing the direct or indirect behavior of the band gap, tends to underestimate the band gap values for semiconductor materials. Thus, in order to enhance the accuracy of our calculated band gap values, we employed the HSE06 functional.

The HSE06 functional has yielded more accurate results when compared to experimental data [3]. Upon applying the HSE06 functional to our calculations, we observed notable increases in the band gap values for both MoSTe and WSTe. Specifically, the band gap energy for MoSTe was determined to be 1.49 eV, while for WSTe, it was calculated to be 1.56 eV, using the HSE06 functional. Importantly, the obtained band gap energies using the HSE method are in good agreement with other theoretical reports [16,26,27].

Figure 2a,b visually demonstrate the increments in the band gap values when using the HSE06 functional. Notably, the indirect behavior of the band gap is preserved, indicating the reliability of both functionals in capturing this characteristic. Furthermore, in the figures, we include the density of states, providing additional insight. The density of states reveals similar behavior between the two functionals, with a shift to higher energy levels in the conduction band.

**Figure 3:**
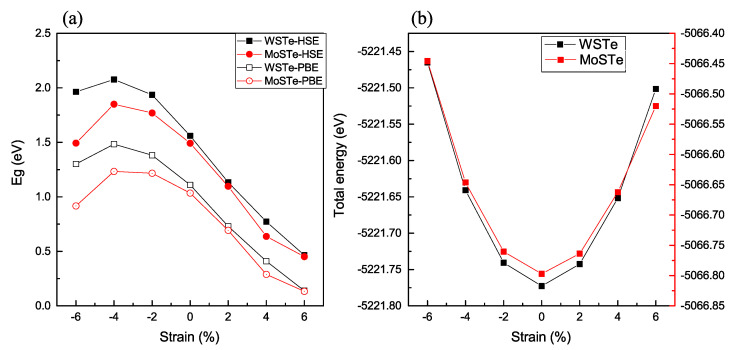
(**a**,**b**) The variation of the band gap and total energy with the strain.

To better understand the impact of strain on the monolayers, we conducted calculations involving tensile and compressive strains. We applied in-plane biaxial tensile and compressive strains, ranging from −6% to 6%. Figure 3 illustrates the relationship between band gap energy and total energy with various strains applied to the WSTe and MoSTe monolayers using the two previously described functionals.

As depicted in Figure 3a, the results from our study demonstrate a clear relationship between strain and the energy band gap of the material. It is evident that the maximum energy of the band gap consistently occurs under a compressive strain of −4%. This finding indicates that the material’s electronic properties are highly influenced by compressive strain, and at this particular strain level, the band gap reaches its most favorable and stable state. Furthermore, our investigations reveal intriguing behavior as we vary the strain beyond −4%. Both tensile and compressive strains impact the band gap, leading to notable changes in the material’s electronic structure. We observe a considerable decrease in the band gap energy with increasing tensile or compressive strain. This decreased band gap with increasing strain can be attributed to the altered interatomic distances and electronic interactions within the material’s crystal lattice. Under tensile strain, the lattice expands, and the spaces between atoms increase. As a result, the energy levels of the electrons within the material shift, leading to a reduction in the band gap. Similarly, under compressive strain, the lattice contracts, causing a narrowing of the band gap. The changes in the band gap due to the compressive strain are not as prominent as in the case of tensile strain; however, they still contribute significantly to the overall behavior of the material. To quantify the sensitivity of the band gap to strain, we compare the slopes of the band gap changes within different strain ranges. Specifically, we compare the −4% to 6% strain range (encompassing both tensile and compressive strains) and the −4% to −6% range (purely compressive strain). Surprisingly, our analysis reveals that the energy band gap is more sensitive to tensile strain, as it exhibits a constant decrease over the strain range. In contrast, the band gap variation under the purely compressive strain remains relatively stable over the same range. This sensitivity to tensile strain suggests that tensile strain significantly impacts the material’s electronic properties more than compressive strain.

Additionally, we examine whether the monolayer could withstand compression or tensile strain by comparing the total energy dependence with strain (Figure 3b). The lowest value of the final energy corresponds to the absence of strain applied to the monolayer. Conversely, when the strain is applied, the distance between the atoms increases or decreases accordingly, resulting in a higher degree of orbital overlap. This increase in the number of electronic bands leads to an overall increment in the total energy of the system.

**Figure 4:**
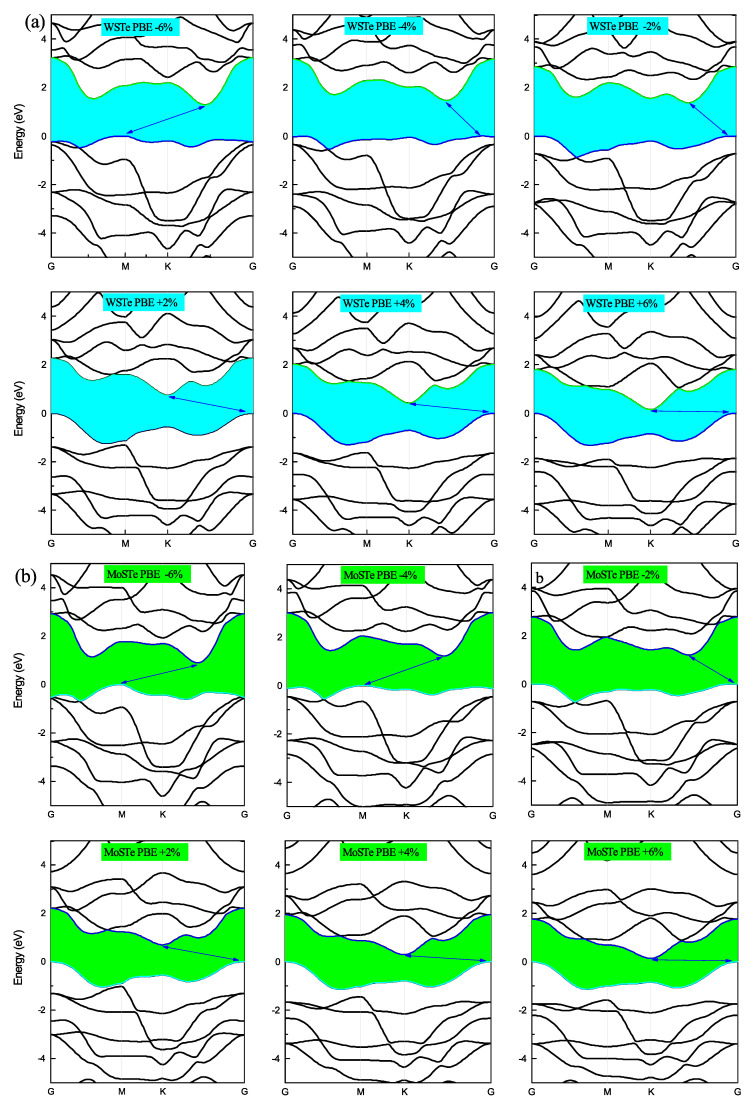
(**a**,**b**) The band structures of MoSTe and WSTe for each strain percentage calculated with PBE.

**Figure 5:**
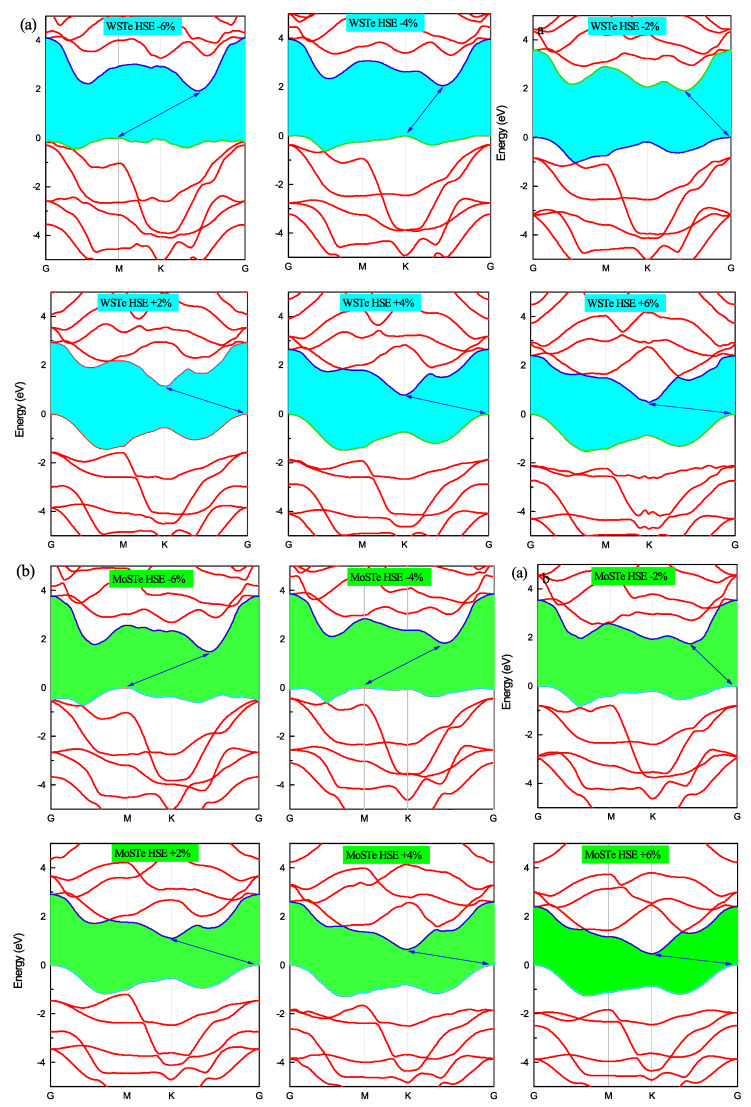
(**a**,**b**) The band structures of MoSTe and WSTe for each strain percentage calculated with HSE.

In Figure 4 and Figure 5, we present the electronic band structures of the WSTe and MoSTe monolayers under different strains. We observe that, under compressive strain, the minimum of the conduction band and the maximum of the valence band remain at high symmetry points, K and Gamma, respectively. However, under tensile strain, the maximum of the valence band shifts from Gamma to K at a strain of −4%, and from K to M at a strain of −6%. The minimum of the conduction band also shifts its position from K to an intermediate location between K and Gamma, as observed in the three band structures at strains of −2%, −4%, and −6%.

We believe that these shifts in the maximum of the valence band and the minimum of the conduction band are caused by variations in the bond strengths between atoms resulting from the compression or tensile of the monolayer. Furthermore, these findings confirm that the band gap is more sensitive to compressive strain than to tensile strain. As the strain increases, the energy band gap of the monolayers decreases, while the band gap tends to increase when the strain decreases.

In Table 1, we present the results of various parameters, including the lattice parameter (*a*), energy band gap ($E_{g}$), deformation potential ($E_{p}$), elastic modulus (*C*), effective masses ($m_{e}^{*}$, $m_{h}^{*}$), electron mobility (μ), relaxation time (τ), and exciton binding energy ($E_{b}$).

The carrier mobility can be determined using the deformation potential (DP) theory, with the following expression:

(2) $$\mu =\frac{2e\hbar^{3}C}{3k_{B}T{\left|m^{*}\right|}^{2}E_{p}^{2}}$$

Here, $E_{p}$ represents the deformation potential, $m^{*}$ denotes the effective carrier mass, *C* signifies the elastic modulus in the strain direction, μ indicates the carrier mobility, and $k_{B}$ represents the Boltzmann constant.

The effective mass is calculated by fitting a parabolic curve to the band edge, given by $m^{*}=\hbar^{2}{\left[\frac{\partial^{2}E\left(k\right)}{\partial k^{2}}\right]}^{-1}$, where E(k) represents the total energy. The deformation potential is defined as the $E_{p}=\frac{\partial E_{\mathrm{edge}}}{\partial \delta }$.

The elastic modulus can be calculated using the equation $C=\frac{1}{A_{0}}\frac{\partial^{2}E_{s}}{\partial \delta }$, where $A_{0}$ is the equilibrium area of the monolayer, and $E_{s}$ represents the strain energy, which is the difference in energy between the strained and unstrained systems ($E_{s}=E_{T_{\delta }}-E_{T_{\delta =0}}$). The strain δ is expressed as a percentage ($\delta =\frac{\Delta a}{a_{0}}$), where $a_{0}$ denotes the lattice constant of the monolayer at the equilibrium state.

Furthermore, we determine the exciton-binding energy using the Wannier–Mott equation: $E_{b}=\frac{m_{e}^{*}m_{h}^{*}R_{y}}{m_{0}\left(m_{e}^{*}+m_{h}^{*}\right)\varepsilon_{0}^{2}}$. Here, $\varepsilon_{0}$ represents the static dielectric constant, $m_{e}^{*}$ and $m_{h}^{*}$ are the average effective masses in all directions, and Ry=13.6057 eV denotes the Rydberg constant.

**Figure 6:**
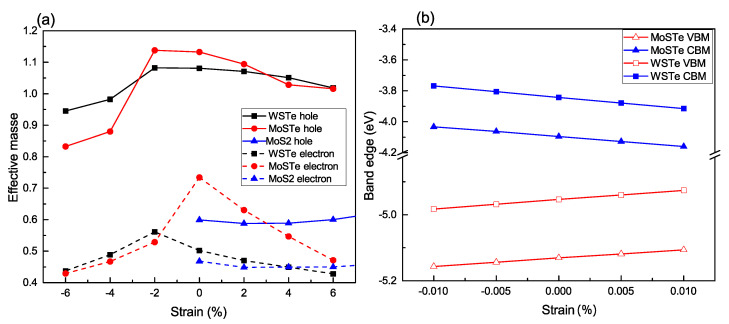
Variation of (**a**) the effective mass and (**b**) the band edges with strain. The data for MoS_2_ are obtained from [28].

According to our results, the calculated effective mass at the equilibrium position is 0.5 $m_{0}$ for WSTe and 0.73 $m_{0}$ for MoSTe. Figure 6a illustrates the dependence of the effective mass under strain ranging from −6% to 6%. We observe that both the electron and hole masses reach their maximum values at a strain of approximately −2%, except for MoSTe, which exhibits its maximum value when no strain is applied. Furthermore, as the strain deviates from the maximum values of the effective masses, both masses tend to decrease.

This behavior can be explained by the deformation of the electronic band structure, resulting in shifts in the positions of both the valence band’s maximum and the conduction band’s minimum. Consequently, the values of the effective masses are altered accordingly.

In our investigation of the Janus structures, we observe a remarkable distinction in the effective masses of electrons and holes compared to conventional transition metal dichalcogenide (TMDC) monolayers, such as MoS${}_{2}$ [28], as can be seen in Figure 3a. The significant differences in effective masses can be attributed to several factors inherent to the Janus structures. Firstly, the presence of the Janus interface plays a crucial role in introducing electronic asymmetry. This interface comprises two distinct chalcogen atoms located on opposite sides of the transition metal atom, inducing changes in the electronic structure, thereby leading to modified effective masses for electrons and holes. Secondly, strain-induced anisotropy is another contributing factor. When strain is applied to the Janus structures, the lattice parameters and bond lengths may undergo different changes along various crystallographic directions. This anisotropic strain response can cause distinct modifications in the electronic states, resulting in different effective masses for electrons and holes along specific directions. The combination of these factors, along with the unique structural and electronic characteristics of Janus structures, give rise to the observed significant differences in effective masses.

In Figure 6b, we depict the variations of the band edges as functions of the applied strains on the Janus monolayers. The elastic moduli of the unstrained monolayers are 105.43 and 112.92 N/C for WSTe and MoSTe, respectively.

**Figure 7:**
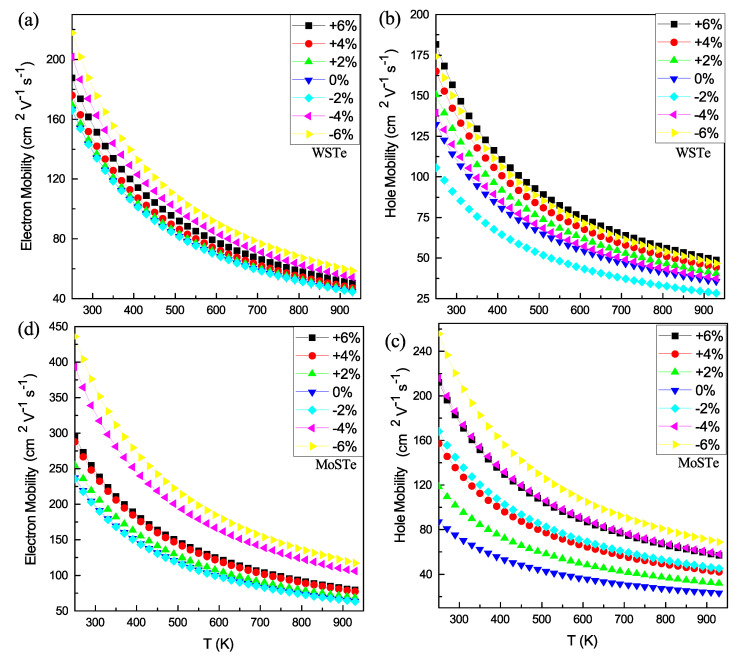
(**a**–**d**) Strain effect on the mobility of MoSTe and WSTe.

In Figure 7, we present the electron (a and c) and hole (b and d) mobilities for WSTe and MoSTe; as observed in both monolayers (Figure 7a,d), the hole mobility surpasses the electron mobility. Additionally, in WSTe and MoSTe monolayers (Figure 7a,d), the hole mobility reaches its maximum value at a strain of −6%, while the minimum is achieved at a 0% or −2% strain. In the case of WSTe (Figure 7b), the electron mobility exhibits its maximum value at a strain of 6% and its minimum at −2%. Similarly, in the MoSTe monolayer, the electron mobility reaches its maximum value at a strain of −6% and its minimum at the equilibrium state.

Based on these findings, we conclude that the hole mobilities of both monolayers and the electron mobility of MoSTe increase with tensile strains of −4% and −6%. The disparity between electron and hole mobilities suggests a lower probability of photo-induced carrier recombination, thereby improving the separation of electron–hole pairs [29]. Additionally, the mobilities decrease with increasing temperature, following an exponential decay pattern in all cases.

**Figure 8:**
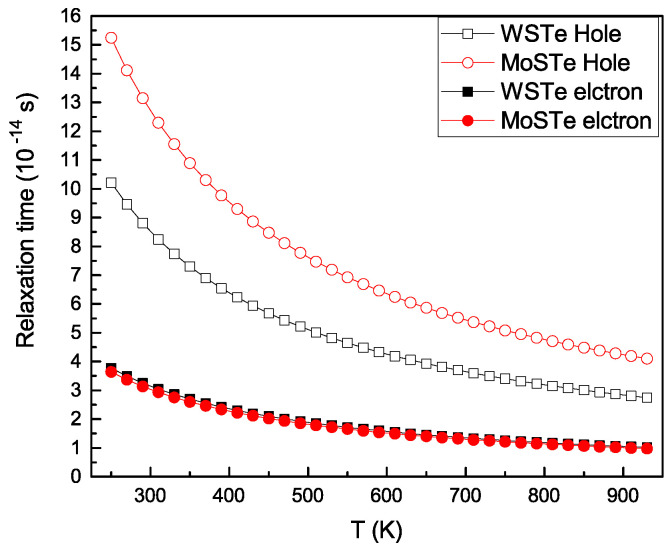
Relaxation time as a function of temperature for MoSTe and WSTe.

In Figure 8, we illustrate the dependence of the relaxation time on temperature. It can be observed that both electrons and holes exhibit decreases in relaxation times as temperature increases. Moreover, the relaxation times for holes decay faster than those for electrons.

When considering the mechanical behaviors of the monolayer materials under strain, the role of defects emerges as a critical factor. While our focus has been on investigating the electronic structure and carrier mobility, it is essential to acknowledge the profound influence of defects in comprehending the material’s mechanical response. Acting as stress concentrators, defects and vacancies give rise to localized strain concentrations, resulting in a diminished overall strength of the material. Even minor defects have been shown to trigger material failure at much lower strain levels than defect-free monolayers. Therefore, a thorough investigation of the impact of defects and their formation energies in monolayer materials is imperative for a comprehensive understanding of their mechanical properties. Moreover, to gain valuable insights into practical applications and material design considerations, future research must delve into the role of defects in the mechanical behaviors of Janus heterostructures. In practical applications, straining a crystal structure up to 6% can present various challenges and limitations that require careful consideration. Structural stability is a primary concern, as excessive strain may lead to instabilities and phase transitions, causing distortions that could lead to material failure. High strain levels can also result in structural damage, such as bond breaking and the formation of defects, significantly altering the material’s properties and affecting its usability. Additionally, materials may exhibit non-linear behavior under high strain, complicating the interpretation of experimental results and property predictions. Anisotropy in crystal structures can further complicate matters, making the material’s response highly direction-dependent. Moreover, materials have elastic limits beyond which they may not fully recover from applied strain, resulting in permanent deformation or structural failure. In applications involving strained materials integrated with substrates, the compatibility of strained materials with surrounding materials is essential to avoid potential interface issues, such as delamination or cracking. By addressing these challenges and considering the impact of defects, a more robust understanding of the mechanical behaviors of strained monolayer materials can be obtained, facilitating their use in diverse applications.

## 4. Conclusions

In conclusion, we investigated the effect of strain on the electronic properties of Janus MoSTe and WSTe. We obtained band gap energies of 1.49 eV and 1.56 eV using the HSE06 hybrid functional for MoSTe and WSTe. These results are in good agreement with the reported data. We observed that the strain significantly impacted the band structure, the bandgap energy, and the effective masses, culminating in a shift in the maximum valance band. Also, our results demonstrate that the Janus monolayers have a band gap that is more sensitive to tensile strain than compression strain, peaking when the strain applied is −4%. Effective masses vary based on the strain applied. This effect could be related to alterations in bond distances, which change the electronic band structure. The carrier mobility in dependence on the temperature reveals that WSTe and MoSTe have very similar behaviors but with a higher value at around 450 ${\mathrm{cm}}^{2}V^{-1}s^{-1}$ for the MoSTe monolayer. Finally, our results demonstrate that when considering relaxation time as a function of temperature, the relaxation time of holes diminishes more rapidly than that of electrons. Still, at lower temperatures, the value related to holes is approximately four times greater than that of electrons.

## Figures and Tables

**Table 1:** Results of all our calculations.

	WSTe	MoSTe		WSTe	MoSTe
Lattice parameter *a* (Å),	3.359	3.381	Electron mobility (${\mathrm{cm}}^{2}V^{-1}s^{-1}$)	70.36	38.02
Band gap with PBE (eV)	1.1	1.03	Hole mobility $\left({\mathrm{cm}}^{2}V^{-1}s^{-1}\right)$	64.57	79.21
Band gap with HSE (eV)	1.56	1.49	Electron relaxation time $(10^{-14}$ s)	1.99	1.59
Electron deformation potential (eV)	−7.34	−46.39	Hole relaxation time ($10^{-14}$ s)	3.97	5.07
Hole deformation potential eV	3.03	2.51	Exciton binding energy (meV)	314.5	407.6
Electron effective masses ($m_{0}$)	0.5	0.73	Elastic modulus (N/m)	105.43	112.92
Hole effective masses ($m_{0}$)	1.08	1.13			

## Data Availability

No experimental data is used in this article.
